# A unique group of scabies mite pseudoproteases promotes cutaneous blood coagulation and delays plasmin-induced fibrinolysis

**DOI:** 10.1371/journal.pntd.0008997

**Published:** 2021-01-06

**Authors:** Deepani D. Fernando, Simone L. Reynolds, Gunter Hartel, Bernard Cribier, Nicolas Ortonne, Malcolm K. Jones, Katja Fischer

**Affiliations:** 1 Cell and Molecular Biology Department, Infectious Diseases Program, QIMR Berghofer Medical Research Institute, Brisbane, Australia; 2 Statistics Unit, QIMR Berghofer Medical Research Institute, Brisbane, Australia; 3 Clinique Dermatologique, Hopitaux Universitaires et Université de Strasbourg, Centres Hospitaliers et Universitaires (CHU) de Strasbourg, Strasbourg, France; 4 Pathology Department, AP-HP, Henri Mondor Hospital and Paris Est Creteil University, Creteil, France; 5 School of Veterinary Science, The University of Queensland, Gatton Campus, Gatton, Australia; Federal University of Ceará, Fortaleza, Brazil, BRAZIL

## Abstract

**Background:**

Scabies, a highly contagious skin disease affecting more than 200 million people worldwide at any time, is caused by the parasitic mite *Sarcoptes scabiei*. In the absence of molecular markers, diagnosis requires experience making surveillance and control challenging. Superficial microthrombi in the absence of vasculitis in scabies-affected skin are a recognised, yet unexplained histopathological differential of scabies infection. This study demonstrates that a family of Scabies Mite Inactivated Cysteine Protease Paralogues (SMIPP-Cs) excreted by the mites plays a role in formation of scabies-induced superficial microthrombi.

**Methodology/Principal findings:**

A series of *in vitro* and *ex vivo* experiments involving two representative recombinant SMIPP-Cs was carried out. In the presence of SMIPP-Cs, the thrombin clotting time (TCT), fibrin formation and plasmin induced fibrinolysis were monitored *in vitro*. The ultrastructure of the SMIPP-C—modulated fibrin was analysed by Scanning Electron Microscopy (SEM). Immuno-histological analyses were performed *ex vivo*, to localise the SMIPP-C proteins within scabies infected skin biopsies. SMIPP-Cs displayed pro-coagulant properties. They bound calcium ions, reduced the thrombin clotting time, enhanced the fibrin formation rate and delayed plasmin-induced fibrinolysis. The SMIPP-Cs associated with fibrin clots during fibrinogen polymerisation and did not bind to preformed fibrin. Scanning electron microscopy revealed that the fibrin clots formed in the presence of SMIPP-Cs were aberrant and denser than normal fibrin clots. SMIPP-Cs were detected in microthrombi which are commonly seen in scabietic skin.

**Conclusions/Significance:**

The SMIPP-Cs are the first scabies mite proteins found in sub-epidermal skin layers and their pro-coagulant properties promote superficial microthrombi formation in scabetic skin. Further research is needed to evaluate their potential as diagnostic or therapeutic target.

## Author summary

Skin-burrowing scabies mites cause pathological changes in the skin. Although these parasites burrow only down to the border between the epidermis and dermis, fibrin thrombi within the dermal vessels are frequently seen in scabies infected skin. Their origin had been unknown. We show by immunohistology that a unique family of mite proteins, the SMIPP-Cs, is found within these fibrin thrombi. We further demonstrate that the SMIPP-Cs promote blood coagulation, change the structure and density of fibrin clots and make them resistant to fibrinolysis.

This may be a novel mechanism by which scabies mites maintain themselves in the microenvironment of the skin. They reside in the lower epidermis close to the dermis to ensure their nutritional requirements are met, and are thereby exposed to the inflammatory and immune responses delivered into the epidermis with the interstitial fluid. To defend themselves from the host immune system the mites release anti-inflammatory, immunosuppressive and, we propose, hemostasis-modulating novel proteins into their microenvironment. Only the scabies mites have evolved the SMIPP-Cs, which appear to play essential roles in the parasitic lifestyle. SMIPP-Cs may present a novel diagnostic or therapeutic target.

## Introduction

Scabies is a common but neglected infectious skin disease caused by the obligate parasitic mite *Sarcoptes scabiei*, affecting hundreds of million people globally [1]. Scabies mites burrow within the host epidermis, causing severe pruritus. Scratching and mechanical damage facilitates the entrance of opportunistic pathogenic bacteria, including *Staphylococcus aureus* and *Streptococcus pyogenes* [2]. Bacterial co-infections can result in pyoderma, post-streptococcal glomerulonephritis [3], rheumatic fever [4] and rheumatic heart disease [1,2,5].

Superficial cutaneous microthrombi in the absence of vasculitis are often seen in scabies skin histology [6] and have been proposed as a diagnostic histopathological feature [6]. Wooten and Gaafar 1984 [7], presumed the presence of a *S*. *scabiei* specific agglutination factor, but the association between microthrombi and active scabies is not well recognised in clinical dermatology and the underlying pathophysiology is unknown. Here we investigate a group of mite proteins for their role in this.

Several classes of mite intestinal excretory proteins are essential to the parasite and co-infecting bacteria for host immune evasion [8–12]. Among these multicopy families, Scabies Mite Inactivated Serine Protease Paralogs (SMIPP-Ss) [13,14] and Serpins (SMSs) [15] interfere with complement-directed killing of bacteria by neutrophils [10,15], and thereby may facilitate scabies-associated bacterial infections [8].

Another intestinal excretory multicopy family are the Scabies Mite Inactivated Cysteine Protease Paralogues (SMIPP-Ca-e) [16]. SMIPP-Cs are homologous to the proteolytically active scabies mite cysteine proteases (Sar s 1a-e) and to the house dust mite group 1 allergens [17], but differ from these in their mutated catalytic sites (Cys-Ser and His-Gln/Leu) [17]. Their sequence homologies suggested that unique structural and functional properties have evolved in all five SMIPP-Cs from an ancestral protease gene, to bring about a new function, different from proteolytic digestion. To elucidate the actual roles of SMIPP-Cs in mite biology and host interactions, we show here that they are present in microthrombi within scabies infected skin and modulate the local host blood coagulation by accelerating fibrin formation, altering the fibrin microstructure and increasing resistance to fibrinolysis.

## Methods

### Ethics statement

The study followed the Animal Care and Protection Act, in compliance with the Australian code of practice for the care and use of animals for scientific purposes, outlined by the Australian National Health and Medical Research Council. Animal work was approved by the Animal Research Ethics Committee of the QIMR Berghofer MRI (QIMRB-P352). The use of shed skin samples from consenting scabies patients was approved by the Human Research Ethics Committee of QIMR MRI (QIMRB-P2159). Diagnostic, anonymized skin biopsies were obtained with verbal formal consent by the patient from the Pathology collections at CHU de Strasbourg and Hôpital Henri Mondor, Créteil.

### Recombinant SMIPP-C production

Full length SMIPP-Ca (AAS93672.1) and SMIPP-Cc (AAS93675.1) sequences were amplified from a cDNA library [18] using specific primers (S1 Table), and cloned with C-terminal His-tag into pET28a+ and pQE9 expression vectors, sequenced and transformed into *E*. *coli* SHuffle cells (NEB, Germany). Native proteins were expressed and extracted under non-denaturing conditions, and purified to >95% purity using Ni affinity chromatography followed by SP-Sepharose size exclusion chromatography using 50mM HEPES, 140mM NaCl and 2% glycerol at pH 7.5 (SMIPP-C buffer).

### Function and structure predictions

The Iterative Threading ASSEmbly Refinement (I-TASSER) [19] server was used to predict the potential structure and functions of the novel SMIPP-C proteins. The 3DLigandSite [20] was used to predict the ligands that have the potential to bind to SMIPP-Cs and to determine possible binding sites. The 3DLigandSite uses the Phyre software to predict the protein structure based on the amino acid sequence. Predicted structures are used to search a structural library for homologues structures with bound ligands.

### Calcium binding assay

Similar to a previously described methodology [21], SMIPP-Ca, SMIPP-Cc or bovine serum albumin (BSA)(1μg) were run on a 10% SDS-PAGE gel and transferred onto a PVDF membrane. The membrane was incubated at room temperature (RT) in 10mM imidazole (pH 6.8), 60mM KCl and 5mM MgCl_2_ for 2hr, rinsed with water and incubated in 1mM CaCl_2_ for 90min, washed with 20% ethanol for 2min, rinsed with water, incubated with 1mM Quin-2 (Sigma Aldrich, USA) for 1hr, rinsed with water and visualised under UV light.

### Blood coagulation tests

Citrated human plasma from two consenting healthy volunteers were pre-tested with normal coagulation parameters. Samples were pooled and mixed with 50mM HEPES and 140mM NaCl at pH 7.5 and SMIPP-C proteins or SMIPP-C buffer (negative control) to a final plasma concentration of 40% and incubated for 10min at 37°C in duplicates. The Thrombin Clotting Time (TCT) was determined in duplicates using the STA—Thrombin kit (Diagnostica Stago, France) and a Sta-R coagulometer (Diagnostica Stago, France). Statistical analysis was performed using a one-way ANOVA and Dunnett’s comparison (JMP v13.1, SAS Institute).

### Fibrin formation assays

Fibrin polymerisation reactions (100μl) were performed in duplicates in 96 well clear flat bottom polystyrene microplates (Corning, USA). Human fibrinogen (Sigma Aldrich, Australia) was pre-incubated with CaCl_2_ in HEPES buffer for 10min at RT and subsequently mixed with human thrombin (Sigma Aldrich, Australia) and SMIPP-C protein or buffer (negative control). The final component concentrations were typically 0.4mg/ml fibrinogen, 0.8nM thrombin, 0–1μM SMIPP-C protein and 20mM CaCl_2_. Fibrin clot formation was evaluated by monitoring the turbidity change at 405nm for 90min at 30°C using a POLARstar optima spectrophotometer (BMG Labtech, Germany). The fibrinogen clotting time, the rate of the fibrin formation and the final absorbance of the reaction were analysed by ANOVA and Dunnett’s multiple comparison.

### Western blot analyses

Fibrin clots formed for 90min at RT in 0.5ml tubes in the presence or absence of 1μM SMIPP-C protein, or SMIPP-C protein was added to the pre-formed fibrin clots. Clots were separated from the supernatant and washed to remove unbound SMIPP-C proteins. Supernatants and clots were resolved under denaturing conditions on 10% SDS-PAGE and analysed by Coomassie blue staining and Western blotting, using anti-SMIPP-C polyclonal murine antibodies [17] at 1:1000 and 1:2000 dilutions for anti-SMIPP-Ca and anti-SMIPP-Cc respectively and the Odyssey system for detection.

### Scanning electron microscopy

Fibrin clots were formed in duplicates in the presence of 0–4μM SMIPP-C protein as described above in a final volume 200μl at 30°C for 2hr. Glutaraldehyde (20μl of a 25% solution, Proscitech, Australia) was added and the clots fixed for 2hr before washing 3 times for 10 min with PBS. Samples were dehydrated, using a graded ethanol series from 20–100% in 10% steps at 10min intervals, and dried in an Autosamdri 815 series B critical point dryer (Tousimis, USA). Samples were mounted onto scanning electron microscopy stubs using double sided carbon tabs and sputter (Proscitech, Australia) and coated with 6nm of iridium using a KQ150T coater (Quorum, UK). A scanning electron microscope (Zeiss, Jena, Germany) operated at 3kV in high vacuum mode with a 30μm aperture was used to capture images with a backscattered detector (Gatan, USA), which were analysed with Digital Micrograph software (Gatan, USA).

### Fibrinolysis assays

To assess fibrinolysis, fibrin formation assays were performed in duplicates, with plasmin being added to the reaction prior to thrombin. Component concentrations were 8nM plasmin, 4nM thrombin, 1mg/ml fibrinogen and 1μM SMIPP-Ca or SMIPP-Cc. Data were analysed by fitting a flexible curve to each fibrinolysis curve, using a knotted spline approach (JMP v13.1, SAS Institute). A spline using 10 knots was fitted to each curve to ensure that all fits exceeded an R^2^ of 95%. The time point associated with the peak measurement was chosen as the value that maximised the spline curve. The trough was chosen as the first point corresponding to the tenth percentile of the spline to the right of the peak.

### SMIPP-C localisation

Paraffin embedded 4μm consecutive sections were used for microthrombi identification by hematoxylin/eosin staining [22], for SMIPP-C protein localisation using a tyramine amplification technique [23] and to probe with pre-immune mouse serum as a negative control. Incubations were carried out at RT and washed with Tris-buffered saline containing 0.05% Tween (TBST) three times, unless stated otherwise. Sections were de-waxed, rehydrated and endogenous peroxidase activity was blocked with 4% H_2_O_2_ for 10min. Slides were rinsed with water, submerged in Diva Decloaker antigen retrieval buffer (Biocare Medical, USA) and microwaved for 45sec at 100% followed by 15min at 10% power. Sections were cooled to RT, blocked with Background Sniper (Biocare Medical, USA) for 20min and probed with SMIPP-C-specific mouse antibody [17] or with pre-immune mouse serum (1:1000) overnight at 4°C and washed. A 20min incubation with anti-mouse secondary antibody MACH1 was followed by washing and a 20min incubation with MACH1 universal HRP probe (Biocare Medical, USA). Slides were probed with FITC labelled tyramide (Perkin Elmer, UK) for 10min, washed and incubated with anti-FITC-HRP labelled antibody (Jackson Immunoresearch, USA) for 30min. Vector NovaRED (Vector Labs, USA) was used as the peroxidase substrate and slides were counterstained with haematoxylin for 1min, dehydrated in graded ethanol, cleared in xylene and mounted with DPX histology slide mounting medium (Sigma-Aldrich, USA). Slides were scanned with an Aperio-AT turbo microscope (Leica Biosystems, Germany) at x10 and x40 magnification. Images were analysed using eSlide manager and ImageScope viewing software (Leica Biosystems, Germany).

## Results and discussion

### SMIPP-Cs bind calcium ions

SMIPP-Ca and SMIPP-Cc proteins (with predicted relative molecular masses of 36,100 and 37,240 Dalton respectively) were expressed in *E*. *coli* and purified under native conditions as soluble proteins, (>95% purity, S1A and S1B Fig).

To aid in assessing the biological function of the SMIPP-C proteins, *in silico* generated structural models (I-TASSER [24] and 3D-Ligand [20]) were utilised. I-TASSER suggested similarities of SMIPP-C with *Fasciola hepatica* cathepsins [19,24,25], among which Cathepsin L2 has the ability to induce fibrin clot formation by cleaving fibrinogen [26]. 3D-Ligand predicted calcium binding sites at positions Glu_278_, His_279_ and Try_282_ in SMIPP-Ca, and Met_275_ and Try_278_ in SMIPP-Cc (S1D and S1E Fig) [20]. The Ca^2+^ binding properties of both SMIPP-Cs were confirmed experimentally, using the calcium ion indicator Quin-2, which has a high selectivity for calcium ions and negligible affinity for potentially competing cations (S1C Fig).

### SMIPP-Cs reduce thrombin clotting time

**Fig 1:**
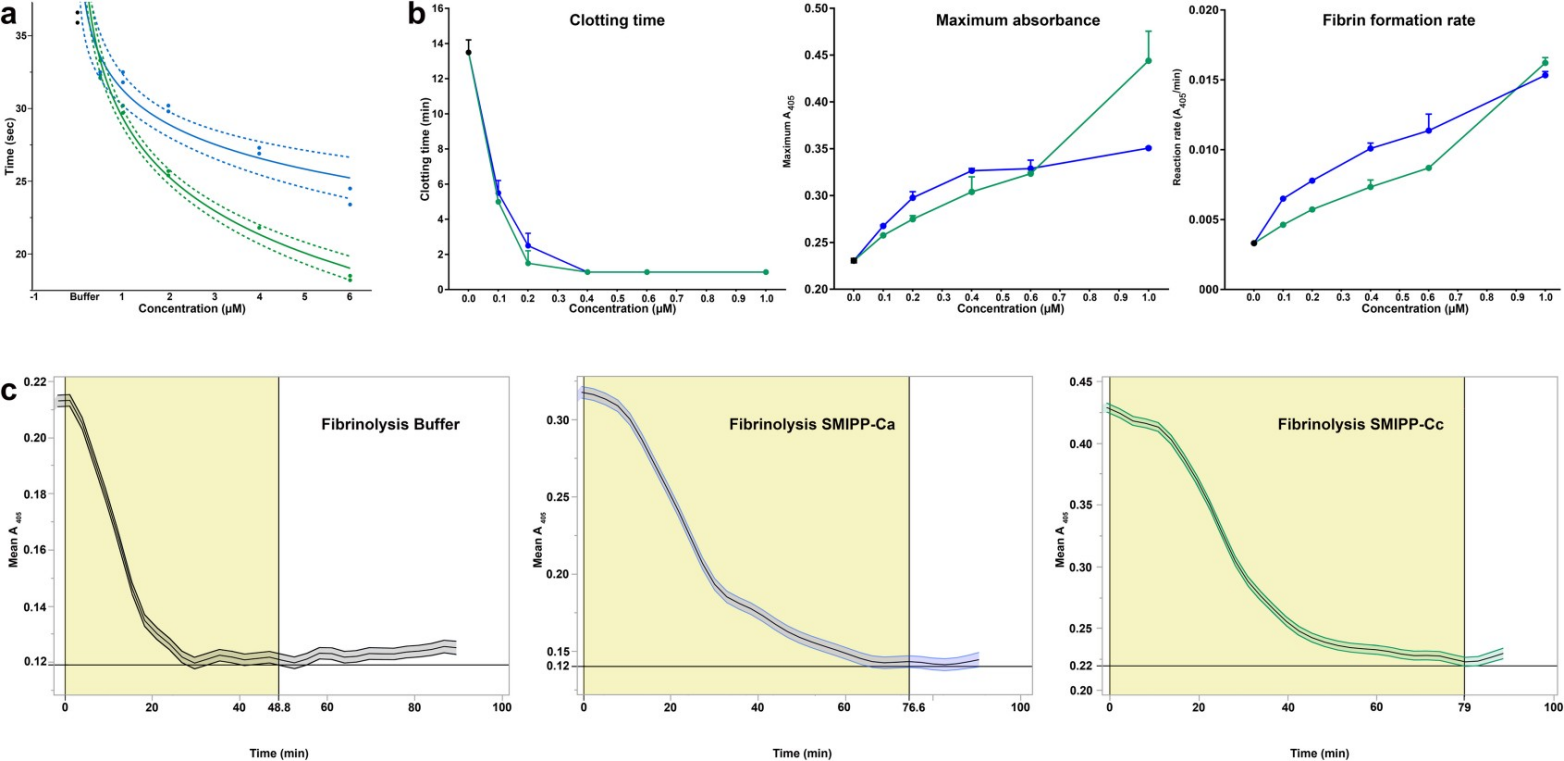
SMIPP-Cs accelerate the fibrin formation and delay plasmin induced fibrinolysis. **(A)** Log-linear relationship of TCT with SMIPP-Ca and SMIPP-Cc. Dashed lines represent 95% confidence bands for the fit of the curve. **(B)** The effect of SMIPP-C proteins on fibrin formation. Based on absorbance data the clotting time, final turbidity and maximum fibrin formation rate in the presence of increasing concentrations of SMIPP-Cs were determined. **(C)** SMIPP-C effect on fibrinolysis. Complete clot lysis with 8nM plasmin was delayed by 35.6 and 36.3min in the presence of 1μM of SMIPP-Ca and SMIPP-Cc respectively. The time from peak to trough was compared between reactions using ANOVA followed by Dunnett’s multiple comparison±SD. Measurements were carried out in duplicate. Black, blue and green represent buffer control, SMIPP-Ca and SMIPP-Cc respectively.

For proof of principle that SMIPP-Cs interfere with clot formation, SMIPP-Cc protein was added to healthy human plasma and tested in a routine diagnostic test (Thrombin Clotting Time, TCT). As previously described by Smith *et al*., 2006 [27], a reduced plasma concentration allowed sensitive detection of clotting. The TCT in healthy undiluted human plasma ranges between 12 and 18sec, and dilution of the plasma to 40% lengthened the TCT approximately two-fold, i.e. 36.25±0.35sec for the buffer control. Compared with the buffer control, both SMIPP-Ca and SMIPP-Cc significantly shortened the TCT in a concentration dependent manner (*P<0*.*001*) (Fig 1A). Regression models estimated that every doubling of SMIPP-C protein concentration reduced the TCT by 2.3±0.2sec for SMIPP-Ca and by 4±0.2sec for SMIPP-Cc.

### SMIPP-Cs enhance fibrin formation

Thrombin–mediated conversion of fibrinogen to fibrin involves protofibril formation, the subsequent lateral aggregation of fibrin fibres, and/or the branching and polymerisation and clot stabilization. The accelerated TCT in the presence of SMIPP-Cs (Fig 1A) suggested that SMIPP-C proteins modulate the common blood coagulation pathway. To investigate this, we performed turbidimetric fibrin formation analysis [28], as previously used to investigate the effects of dermatopontin [29] and polyphosphate [28]. The turbidimetric raw data obtained (S2 Fig) was used to calculate the clotting time, defined as the first time point at which the curve left the baseline [30], the final clot turbidity, represented by the level of the curve plateau, and the maximum fibrin formation rate, calculated as the steepest part of the curve versus time (Fig 1B). Both SMIPP-Cs decreased the clotting time significantly vs buffer (*P<0*.*0001)* in a dose-dependent manner (Fig 1B) from 20min to <1min, indicating increased protofibril formation. This contrasts with dermatopontin [29] and polyphosphate [28] which did not have significant effect of clotting time. The final clot turbidity increased with added SMIPP-Cs (Fig 1B) signifying greater polymerisation of the fibrin formed as with dermatopontin [29] and polyphosphate [28]. The maximum fibrin formation rate (absorbance/min) (Fig 1B) increased in a dose-dependent manner (*R*^*2*^ *= 94%* for SMIPP-Ca and *96%* for SMIPP-Cc), indicating the SMIPP-Cs accelerate fibrin formation possibly by amplified polymerisation and/or branching of the protofibrils. Unspecific protein aggregation is unlikely the reason for the greater absorbance seen in the fibrin formation assay, as the addition of the same amount of Lectin as an unrelated but easily agglutinated protein showed no increased absorbance (S2 Fig). There were also no changes to the absorbance reading when fibrinogen alone, thrombin alone, or SMIPP-C with either fibrinogen or thrombin alone were tested under the same conditions, indicating that SMIPP-Cs do not act on fibrinogen or thrombin alone. Consequently, SMIPP-Cs do not initiate, but enhance coagulation, initiated in scabetic skin by an unknown mechanism. In contrast, dermatopontin interacts with both isolated fibrinogen or fibrin [29], while *Fh* cathepsin L2 interacts only with fibrinogen [26].

### SMIPP-Cs associate with fibrin clots

**Fig 2:**
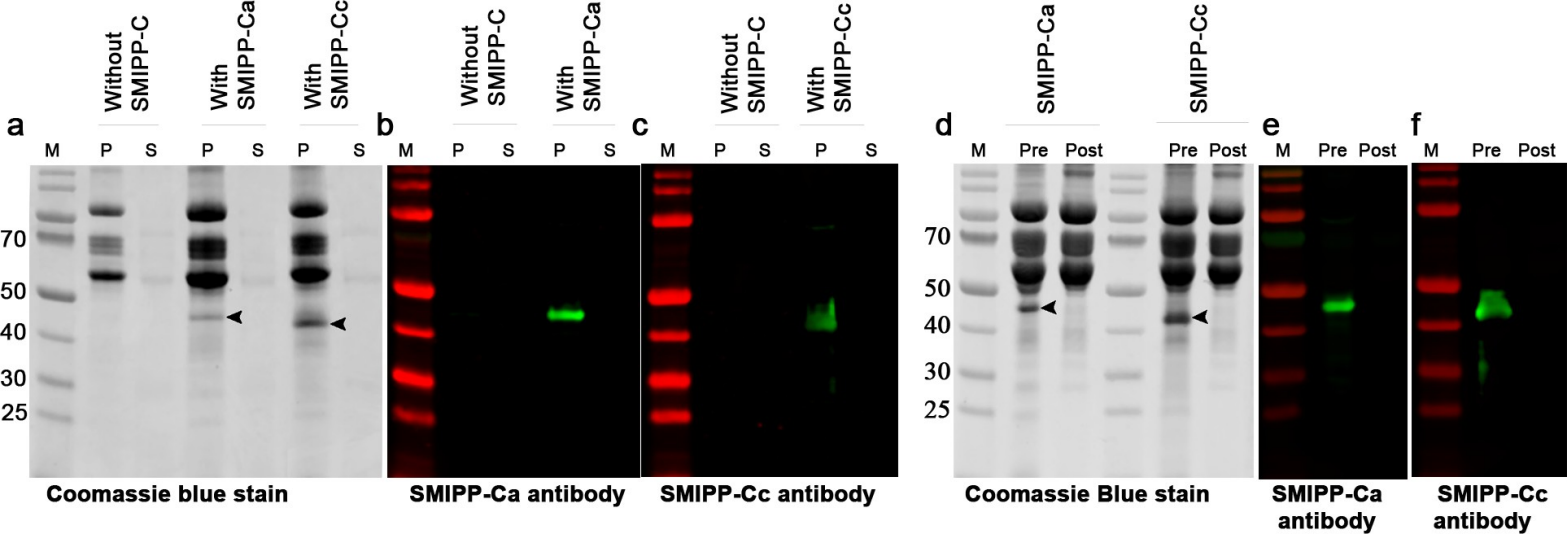
SMIPP-C proteins associate with the fibrin clot during polymerisation and do not bind to pre-formed fibrin. Coomassie blue stained SDS-PAGE gels are shown in **(A)** and **(D)**, and Western blots in **(B)**, **(C)**, **(E)** and **(F)**. SMIPP-Cs associated with the fibrin clot (**A, B, C**: lanes P) leaving no SMIPP-C protein in the supernatant of the reaction (**A, B, C**: lanes S). SMIPP-Cs were associated with the clot only when added before fibrinogen polymerisation (**D**, **E**, **F**: lanes “Pre”). They did not incorporate into already polymerised fibrin (**D**, **E**, **F**: lanes “Post”). M: molecular weight marker; P: fibrin clot pellet; S: fibrin clot supernatant; Arrows: indicate SMIPP-C protein.

To further elucidate the nature of the SMIPP-C interaction with fibrinogen, fibrin clots formed in the presence of SMIPP-Cs, control clots and assay supernatants were subjected to SDS-PAGE electrophoresis and Western blot analysis (Fig 2). SMIPP-Cs were only detected in clots, and not in the corresponding supernatant (Fig 2A). SMIPP-Cs associated with the clot only when they were added prior to the onset of fibrin polymerisation (Fig 2D). In contrast to dermatopontin [29], SMIPP-Cs did not interact with polymerised fibrin (Fig 2D). Western blots probed with SMIPP-C-specific antibodies confirmed these results (Fig 2B, 2C, 2E and 2F). From these data, it seems obvious that the SMIPP-C proteins associate or integrate with the polymerising fibrin clot, but at this stage, it is unclear how this results in increased protofibril formation.

### SMIPP-Cs affect clot structure

Molecules that enhance fibrin formation may have an effect on the structure of fibers in the fibrin matrix. In the presence of dermatopontin [29] or compounds such as polyphosphate [28], dextran, acetylsalicylic acid, poloxamer 188 or hydroxethyl starch, (reviewed in [31]) fibrin fiber thickness increased relative to increasing fibrin clot turbidity, while poly-aspartate or poly-glutamate had no effect on fibrin fiber thickness [31]. We employed SEM visualisation to trace the effects of SMIPP-C proteins on fiber thickness during clotting.

**Fig 3:**
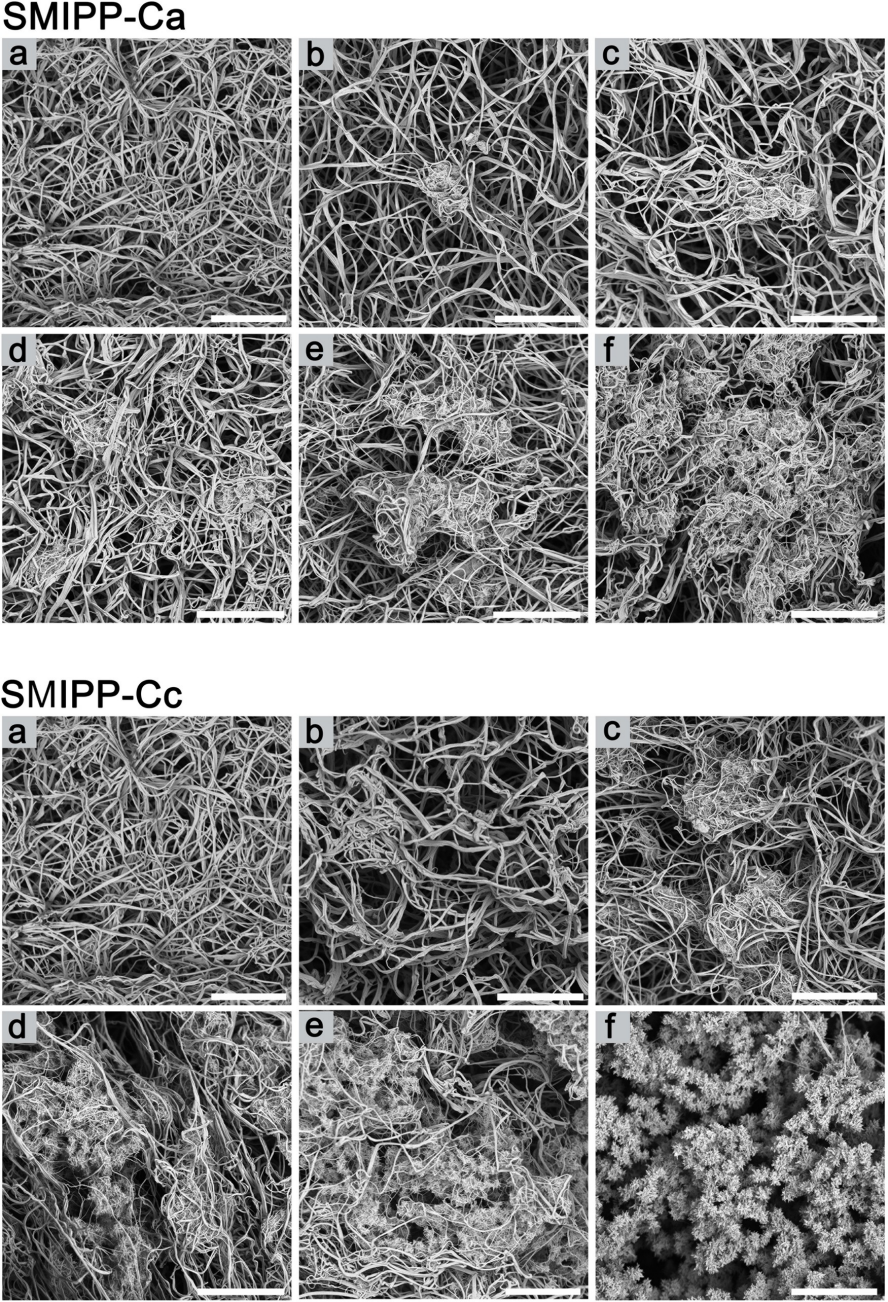
Aberrant fibrin fibril formation in the presence of SMIP-Cs. **(A-F)** Representative scanning electron micrographs of fibrin clots in the presence of no SMIPP-Cs **(A)**, 0.25μM **(B)**, 0.5μM **(C)**, 1.0μM **(D)**, 2.0μM **(E)** and 4.0μM **(F)**. SMIPP-Ca is shown at the top, and SMIPP-Cc at the bottom. Scale bar represents 5μm.

Fibrin clots formed with SMIPP-C buffer added (Fig 3A) showed the expected architecture of fibrin fibrils, consisting of branching arrays of interlocked fibres of uniform diameter [32]. Fibrin clots formed in the presence of SMIPP-Cs showed no obvious change in fiber thickness (S3 Fig) but the clot had a complex, atypical morphology (Figs 3 and S4A–S4D). At low SMIPP-C concentrations, small knots were observed, which became progressively tangled and clustered as the concentration of SMIPP-C increased (Figs 3B–3E and S4A–S4D). At high protein concentrations, the fibrin fibrils were relatively less abundant and were replaced at the knots with feathery projections (Figs 3F, S4E and S4G). SMIPP-Ca, which is less active in fibrin formation than SMIPP-Cc, induced less feathery projections in the clot. Importantly, the SMIPP-C proteins did not aggregate in the reaction buffer or with the pre-formed fibrin at these concentrations.

At higher magnification, small projections from fibrin fibers were observed at low SMIPP-C concentrations (S4A and S4F Fig). As SMIPP-C concentrations rose, multiple fibers formed web-like structures (S4B Fig) that congregated into knots, and further expanded into nest-like structures (S4C Fig), with multiple tangled fibers (S4D Fig). Fibrin fiber alterations often involve changes in fiber thickness [33], but other types of modulations have been described [34,35], however, the complex features induced by SMIPP-C proteins have not been reported before.

### SMIPP-Cs delay plasmin-induced fibrinolysis

Fibrinolysis is highly dependent on fibrin fiber structure and induced by plasmin [33,36], a fibrinolytic enzyme formed from plasminogen through the action of either tissue plasminogen activator (tPA) or urokinase (uPA). The effect of SMIPP-Cs on fibrinolysis was assessed by adding plasmin into the fibrin formation reaction. Different thrombin concentrations were pre-tested to ensure that the maximum absorbance (peak) in all the reactions was achieved at similar times, (20.5min for SMIPP-Ca and buffer control, 20.8min for SMIPP-Cc). All reactions ultimately returned to their starting absorbance, indicating complete lysis of the fibrin clot. The buffer control reaction took 30.9min for complete clot lysis, while 1μM SMIPP-Ca and SMIPP-Cc caused significant delay in fibrinolysis, i.e. 66.5min and 67.2min respectively (Fig 1C, *P<0*.*01* for each comparison). It has been shown that increased fiber thickness negatively affects tPa-mediated plasmin generation [37] and the rate of fibrinolysis [38]. In addition, a tightly packed three-dimensional clot structure is thought to limit access of plasmin to cleavage sites and hence reduces the rate of plasmin activity [36–38]. We propose that the fibrin structure produced in the presence of SMIPP-C protein was resistant to plasmin-induced fibrinolysis because the plasmin binding sites were either altered or obstructed. Alternatively, the rate of fibrinolysis could also have been effected by changes in porosity of the clot, slowing the diffusion of lytic proteins.

### SMIPP-Cs co-localise with microthrombi in scabetic skin

Since the SMIPP-C proteins enhanced fibrin formation and blood coagulation *in vitro*, we hypothesized that we would be able to detect SMIPP-Cs *ex vivo* within superficial microthrombi in scabietic skin. Microthrombi induced by secondary bacterial infections in scabies are usually associated with vasculitis, and histological features are inflammatory cells within the capillary vessel walls, endothelial swelling, capillary wall thickening and extravasation of erythrocytes. To demonstrate the involvement of SMIPP-Cs, we focussed on microthrombi present in the absence of signs of vasculitis. Skin biopsy samples from 16 clinically diagnosed scabies patients of different ethnicity, gender and age (40–83) with no known other health issues were analysed by two dermatopathologists. Scabies mite parts and feaces were observed in heamatoxylin/eosin stained sections from these biopsies (S5 Fig). In addition the presence of eosinophilic, fibrinous to homogenous material, partially to completely obscuring the micro-vessels in the superficial dermis clearly identified as cutaneous microthrombi without evidence of vasculitis (S5 Fig).

**Fig 4:**
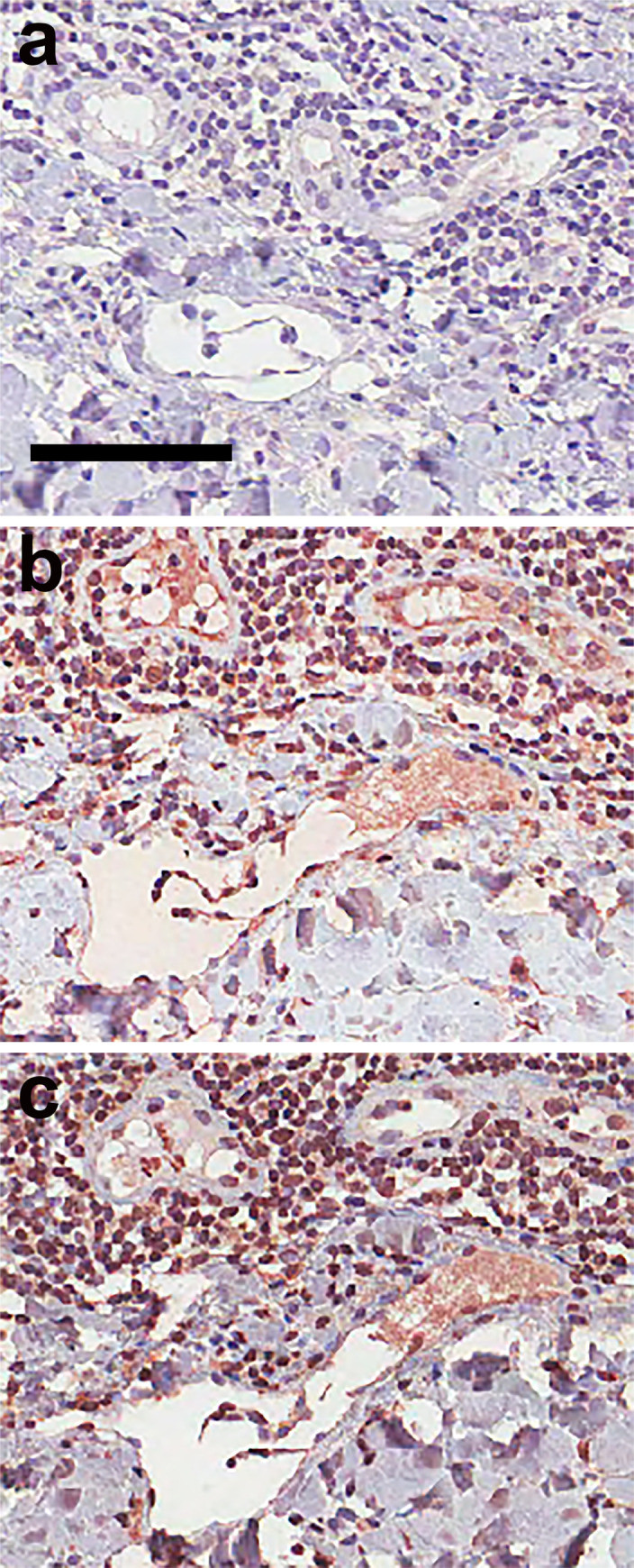
Immunohistological localisation of SMIPP-Cc protein in the microthrombi of scabietic skin. Serial histological sections of blood vessels (A-C) within the dermis of scabies mite infested human skin are shown. Section (A), probed with pre-immune mouse serum as a negative control, remained unstained, whereas sections (B) and (C), probed with antibodies raised against SMIPP-Ca and SMIPP-Cc proteins respectively, stained corresponding regions red. The anti-SMIPP-C antibodies used here have been previously shown to detect SMIPP-C proteins in the mite gut and faeces [17]. Red staining indicates binding of antibody to protein. Scale bars equal 100μm.

Samples from five patients contained microthrombi in consecutive sections, allowing comparative immunohistochemistry with anti-SMIPP-Ca and SMIPP-Cc specific mouse antibodies. Sections probed with pre-bleed serum (negative control) showed no red staining (Fig 4A), while microthrombi in SMIPP-C antibodies probed sections stained red (Fig 4B and 4C). The same antibodies used here were recently shown to detect SMIPP-C proteins within the mite gut and in mite faeces within the epidermis [17].

Earlier work [6,39,40] confirms the occurrence of microthrombi in the skin of scabies patients in the absence of vasculitis and hypothesised that mite proteins were involved, but the identity of these proteins was previously not known.

Why have the parasitic scabies mites, but not the closely related free-living mite species, evolved the multi-copy protein families of SMIPP-Ss [41], SMSs [15] and SMIPP-Cs [16]? What is the evolutionary advantage? With the human coagulation and complement cascades interconnecting at multiple points [42,43], we propose that the anti-complement activities of the SMIPP-Ss and SMSs [13,15] as well as the here reported pro-coagulant activity of the SMIPP-Cs form an essential host immune evasion machinery required by the mite for its parasitic lifestyle [9]. Upon activation, the coagulation cascade generates a three-dimensional protective scaffold for platelets, blood cells and plasma components. It is tempting to speculate that the scabies mite accelerates this system in order to confine itself from the early immune response. Inflammatory cells and immune-reactive proteins (such as antibodies and complement factors) may be trapped and less likely to get into the mite. To accomplish this, we propose that the SMIPP-C proteins diffuse below the mite burrow (Fig 4), and are indeed the first scabies mite molecules to be detected deeper than the epidermis. This localisation of the SMIPP-Cs is consistent with their proposed role in altering fibrin formation, delaying fibrinolysis and contributing to the formation of the microthrombi.

We provide here initial functional data about a novel and unique family of secretory/excretory scabies mite proteins. SMIPP-Cs bind calcium ions, promote blood coagulation, accelerate fibrin formation and alter the natural fibrin structure in complexity and density, thereby delaying plasmin-induced fibrinolysis. Confocal microscopy and/or atomic forced microscopic imaging of SMIPP-C enhanced fibrin clots in association with functional *in-vivo* studies in the porcine scabies model may be used to consolidate the proof of concept data reported here and to appreciate the unabridged physiological role of these proteins.

## Supporting information

S1 Table SMIPP-C specific primers. Primer names and sequences are listed in the left and right column, respectively.
(DOCX)

S1 Fig Characterisation of two recombinant SMIPP-Cs. Purified recombinant SMIPP-Ca **(A)** and SMIPP-Cc **(B)** were separated by SDS-PAGE and detected by Coomassie blue staining and by Western blotting using protein specific mouse polyclonal antibodies. **(C)** SMIPP-C proteins and BSA were separated by SDS-PAGE, transferred to a PVDF membrane and subjected to Quin-2 staining. Calcium-binding SMIPP-C proteins and BSA protein appeared as white fluorescence bands under UV light, while the protein marker (M) appeared black, indicating the absence of Ca^2+^ binding. **(D, E)** Proposed molecular structures of SMIPP-Ca and SMIPP-Cc, including the sites of potential binding residues (blue) for calcium ions (green), as predicted by 3DLigand software.
(TIF)

S2 Fig Turbidimetric raw data showing the effect of (A, B) SMIPP-C proteins and (C) Lectin on the fibrin clotting time, final clot turbidity and maximum fibrin formation rates. The absorbance at 405nm was monitored in an in vitro human fibrin formation assay in the presence of increasing concentrations of SMIPP-Ca (A, blue), SMIPP-Cc (B, green), Lectin (C, orange) compared to the buffer control (black). Each curve is representative of two repeats; error bars represent the standard deviation from the mean.
(TIF)

S3 Fig SMIPP-Cs have no effect on the fibrin fiber thickness. Fibrin clots formed in the presence of SMIPP-Ca (blue), SMIPP-Cc (green) or buffer (black) were imaged at ×5000 magnification by scanning electron microscopy. Two independent areas per image were analysed. A 16 square grid was overlayed over each area and 4 squares were randomly picked following computer generated numbers. The widths of 10 fibers per square were measured with total of 80 measurements per condition. No statistical differences in diameters were observed. Fiber thickness data was analysed using a likelihood ratio test, followed by a Dunnett’s multiple comparison test. The least squares (LS) means and adjusted 95% confidence intervals for each sample type were calculated and plotted using JMP Pro (V15.1, SAS Institute, Cary, NC, USA).
(TIF)

S4 Fig Features of aberrant fibrin fibers formed in the presence of increasing concentrations of SMIPP-Cs. Fibrin fibers with tiny projections (**A**– 0.5μM SMIPP-Cc), knot formation (**B**—0.25μM SMIPP-Ca), nest formation (**C**—0.5μM SMIPP-Cc), fiber tangling (**D**—1μM SMIPP-Cc), feathery fibrin structures (**E**—2μM SMIPP-Cc, **F and G**—4μM SMIPP-Cc). Scale bar 5μm in panels A, B, C, D and E, and 2μm in F and G.
(TIF)

S5 Fig Microthrombi in scabietic human skin. Hematoxylin/eosin (H&E)-stained slides of skin biopsies from five scabies patients. Multifocal superficial vascular thrombi (arrows) in the absence of vasculitis and inflammation were observed in dermal vessels located in proximity to *S*. *scabiei* var. *hominis* mites (m), eggs (e) and mite faeces (f) within epidermal mite burrows (b). Scale bar represents 100μm.
(JPG)
