# Supplementary material for: A unique group of scabies mite pseudoproteases promotes cutaneous blood coagulation and delays plasmin-induced fibrinolysis
Source: PLoS Negl Trop Dis. 2021 Jan 6;15(1):e0008997. doi: 10.1371/journal.pntd.0008997 (PMC7815109; doi:10.1371/journal.pntd.0008997)
Supplement: S1 Table — Primer names and sequences are listed in the left and right column, respectively. (DOCX) [file pntd.0008997.s001.docx]

Supplemental Table 1: SMIPP-C specific primers

| SMIPP-ca pET28aF | ACCGCCATGGCTCaagaattgactgaatctcctccg |
| --- | --- |
| SMIPP-ca pET28aR | ACCGGCGGCCGCTTAATGATGATGATGATGATGGAATTCAGGTCGACCCAATCTGAC |
| SMIPP-cc pQE9F | ACCGGTCGACTATTATTTCGAGACAACGCCAAGTGATGCTG |
| SMIPP-cc PQE9R | ACCGCTGCAGTCATTCAAAATCTTCAGGCTCATTTTCAAAAGG |
