# Supplementary figures and images for: A unique group of scabies mite pseudoproteases promotes cutaneous blood coagulation and delays plasmin-induced fibrinolysis
Source: PLoS Negl Trop Dis. 2021 Jan 6;15(1):e0008997. doi: 10.1371/journal.pntd.0008997 (PMC7815109; doi:10.1371/journal.pntd.0008997)

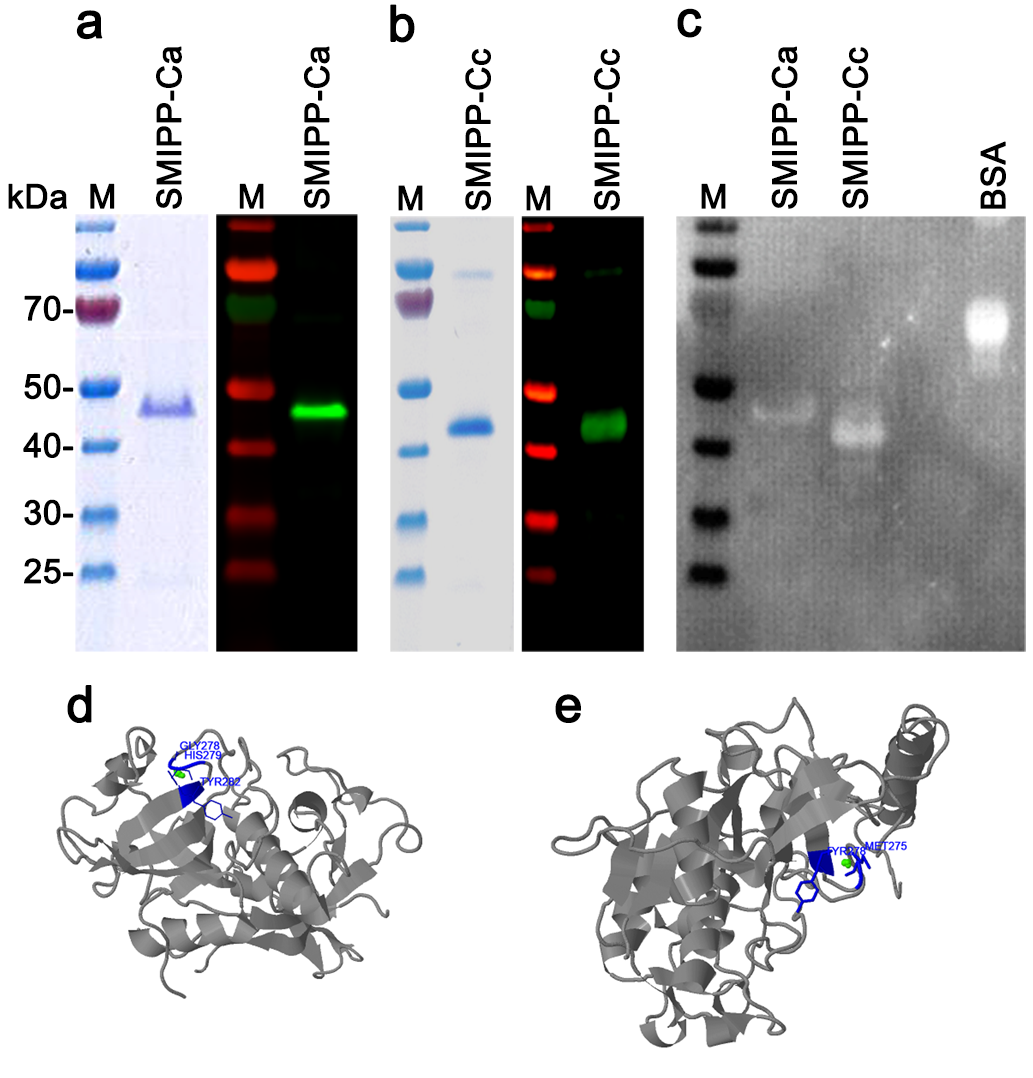

Supplement: S1 Fig — Purified recombinant SMIPP-Ca (A) and SMIPP-Cc (B) were separated by SDS-PAGE and detected by Coomassie blue staining and by Western blotting using protein specific mouse polyclonal antibodies. (C) SMIPP-C proteins and BSA were separated by SDS-PAGE, transferred to a PVDF membrane and subjected to Quin-2 staining. Calcium-binding SMIPP-C proteins and BSA protein appeared as white fluorescence bands under UV light, while the protein marker (M) appeared black, indicating the absence of Ca2+ binding. (D, E) Proposed molecular structures of SMIPP-Ca and SMIPP-Cc, including the sites of potential binding residues (blue) for calcium ions (green), as predicted by 3DLigand software. (TIF) [file pntd.0008997.s002.tif]

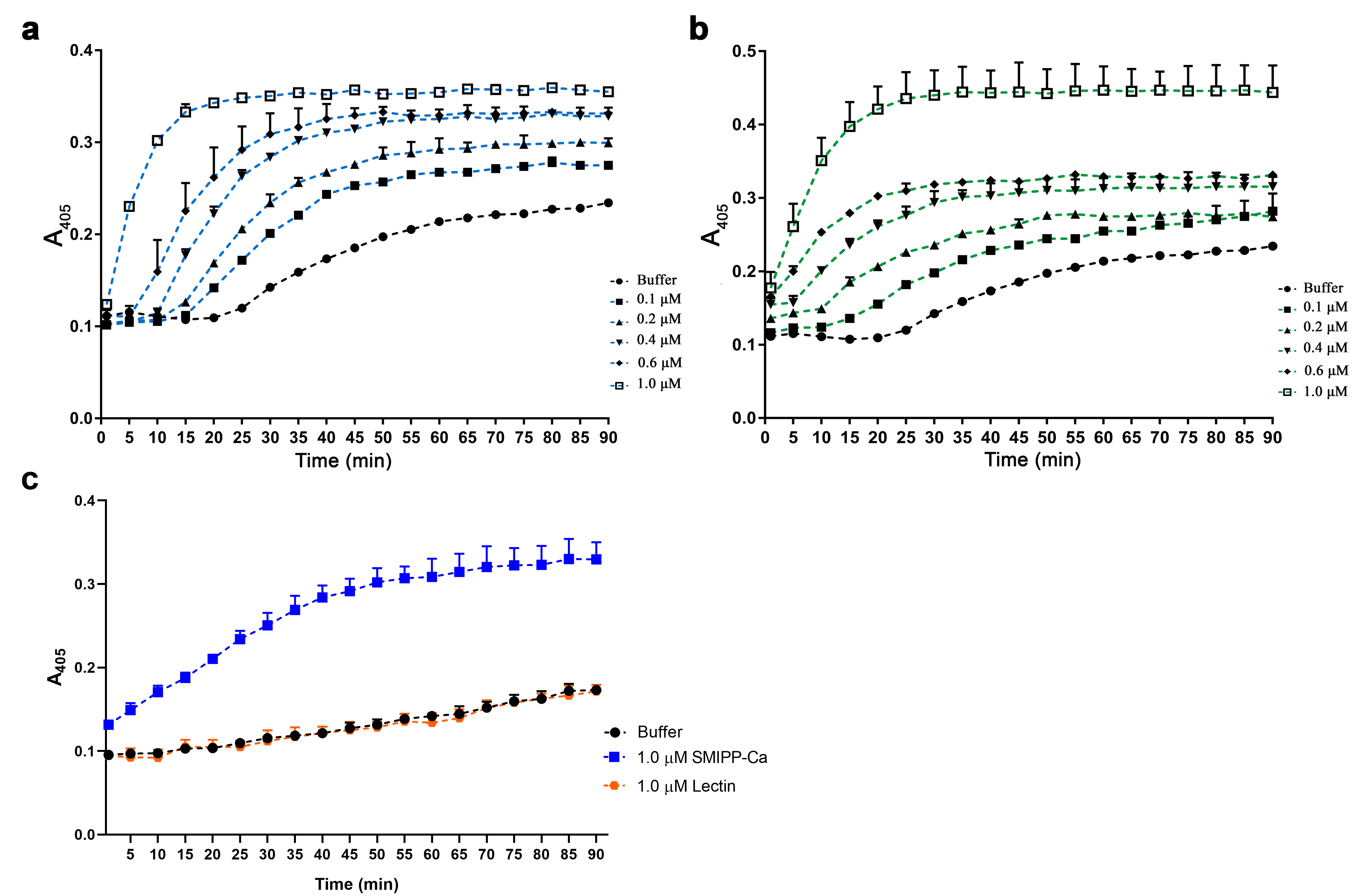

Supplement: S2 Fig — Turbidimetric raw data showing the effect of (A, B) SMIPP-C proteins and (C) Lectin on the fibrin clotting time, final clot turbidity and maximum fibrin formation rates. The absorbance at 405nm was monitored in an in vitro human fibrin formation assay in the presence of increasing concentrations of SMIPP-Ca (A, blue), SMIPP-Cc (B, green), Lectin (C, orange) compared to the buffer control (black). Each curve is representative of two repeats; error bars represent the standard deviation from the mean. (TIF) [file pntd.0008997.s003.tif]

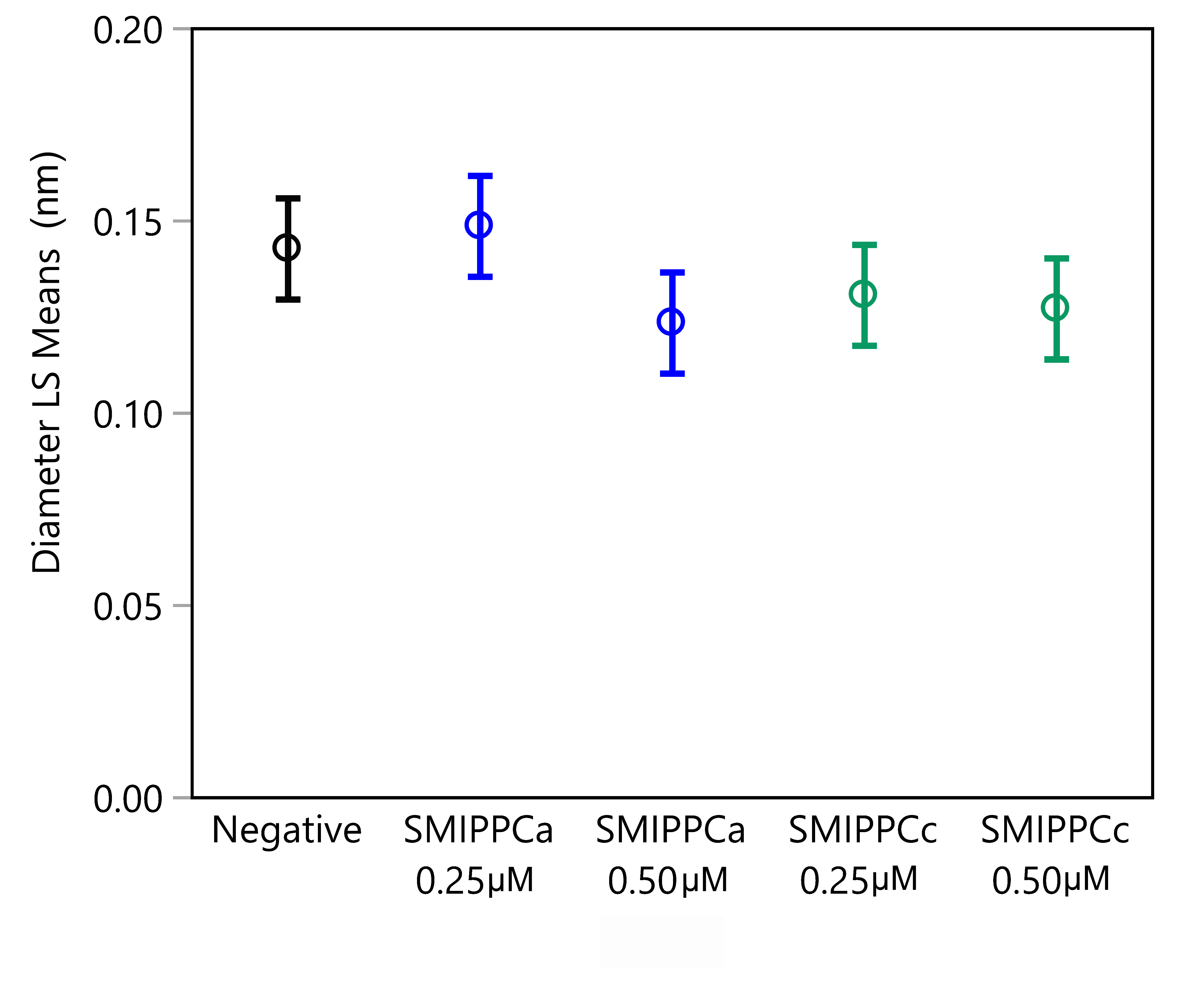

Supplement: S3 Fig — Fibrin clots formed in the presence of SMIPP-Ca (blue), SMIPP-Cc (green) or buffer (black) were imaged at ×5000 magnification by scanning electron microscopy. Two independent areas per image were analysed. A 16 square grid was overlayed over each area and 4 squares were randomly picked following computer generated numbers. The widths of 10 fibers per square were measured with total of 80 measurements per condition. No statistical differences in diameters were observed. Fiber thickness data was analysed using a likelihood ratio test, followed by a Dunnett’s multiple comparison test. The least squares (LS) means and adjusted 95% confidence intervals for each sample type were calculated and plotted using JMP Pro (V15.1, SAS Institute, Cary, NC, USA). (TIF) [file pntd.0008997.s004.tif]

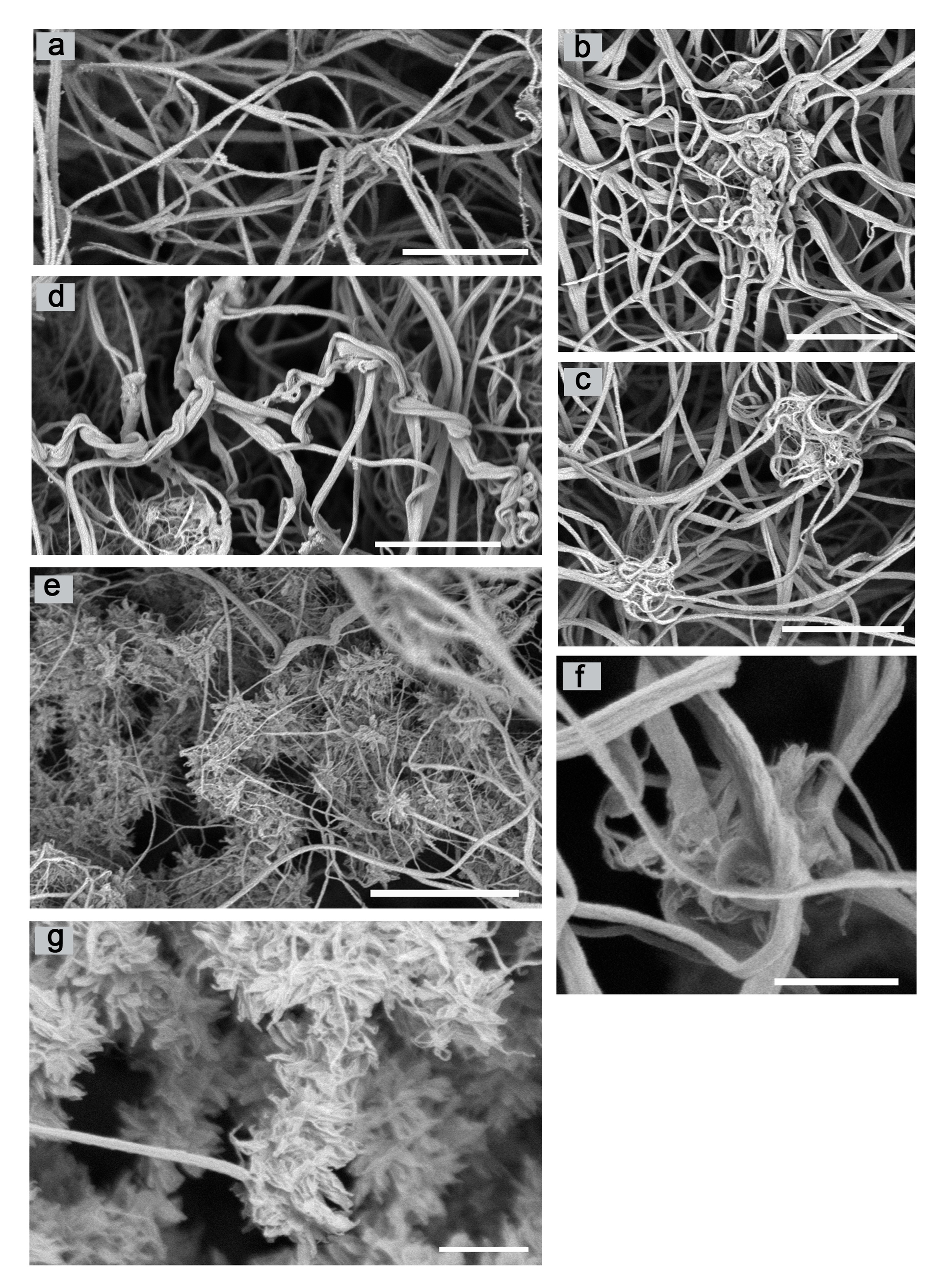

Supplement: S4 Fig — Fibrin fibers with tiny projections (A– 0.5μM SMIPP-Cc), knot formation (B—0.25μM SMIPP-Ca), nest formation (C—0.5μM SMIPP-Cc), fiber tangling (D—1μM SMIPP-Cc), feathery fibrin structures (E—2μM SMIPP-Cc, F and G—4μM SMIPP-Cc). Scale bar 5μm in panels A, B, C, D and E, and 2μm in F and G. (TIF) [file pntd.0008997.s005.tif]

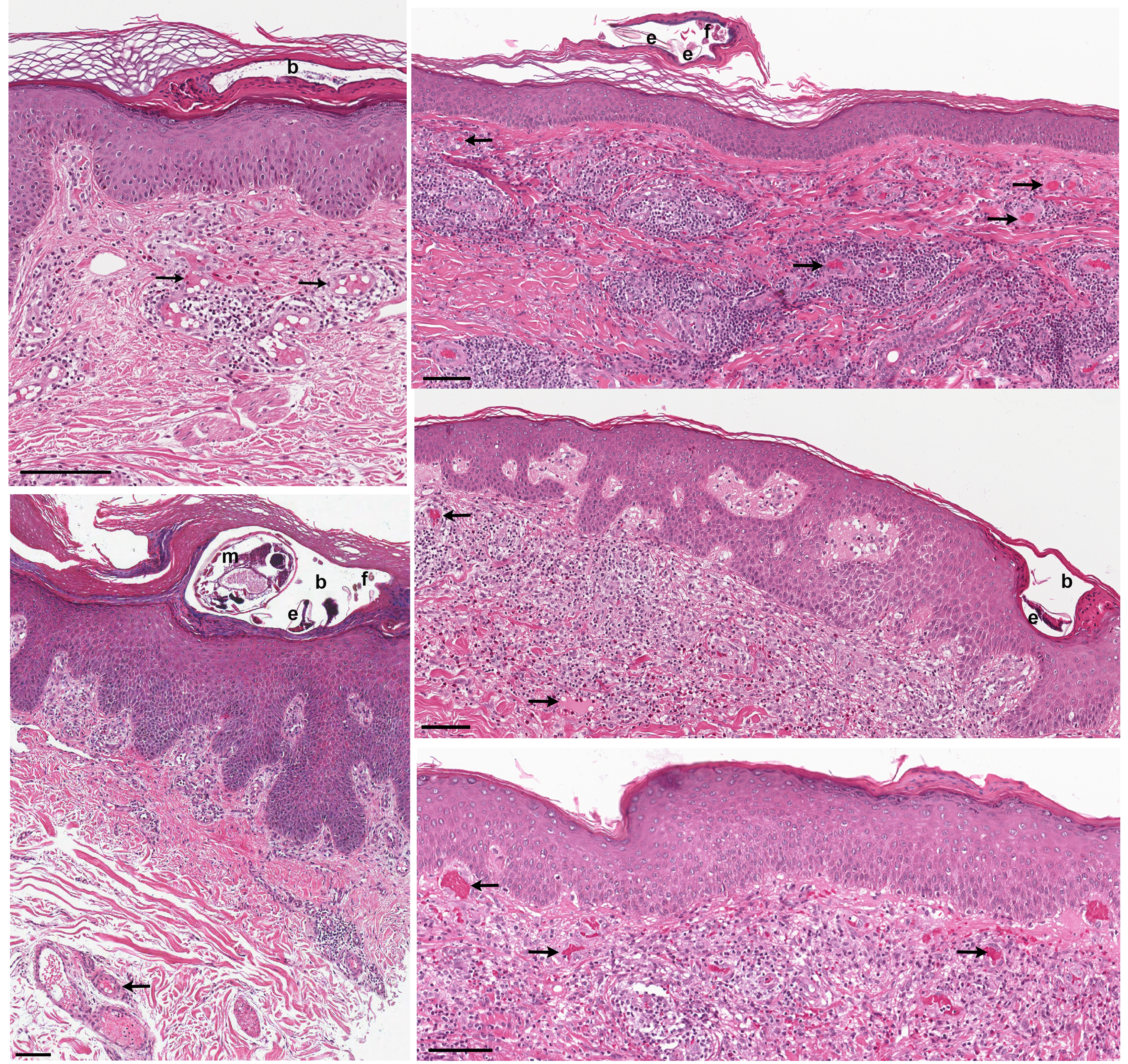

Supplement: S5 Fig — Hematoxylin/eosin (H&E)-stained slides of skin biopsies from five scabies patients. Multifocal superficial vascular thrombi (arrows) in the absence of vasculitis and inflammation were observed in dermal vessels located in proximity to S. scabiei var. hominis mites (m), eggs (e) and mite faeces (f) within epidermal mite burrows (b). Scale bar represents 100μm. (JPG) [file pntd.0008997.s006.jpg]
